# Exogenous Nutrient Bag Formulations Affect Soil Fertility and Microbial Communities in *Morchella sextelata* Cultivation

**DOI:** 10.3390/biology15171505

**Published:** 2026-09-02

**Authors:** Li Gong, Le Wang, Liping Su, Wei Sa, Quanmin Dong

**Affiliations:** 1 Research Institute of Science and Technology, Qinghai University, Xining 810016, China; gongli@qhu.edu.cn (L.G.);; 2 Key Laboratory of Adaptive Management for Alpine Grassland Ecosystem of Qinghai Province, Qinghai University, Xining 810016, China

**Keywords:** exogenous nutrient bags, morel mushroom, physicochemical properties, microbial community analysis

## Abstract

The application of exogenous nutrient bags (ENBs) is critical for achieving high yields in morel mushroom cultivation; however, the high cost and lack of locally adapted formulations have become major constraints for the sustainable expansion of this industry. This study aimed to evaluate the effects of ten treatments, including a conventional formulation control, incorporating locally available agricultural by-products (rapeseed straw, organic fertilizer, alfalfa, and oats), on soil fertility, enzyme activity, microbial community structure, and ultimately the yield and quality of cultivated *Morchella sextelata* in Jianzha County, Qinghai. A randomized complete block design was established, and soil physicochemical properties, microbial biomass carbon/nitrogen/phosphorus (MBC/MBN/MBP), and enzyme activities (urease, phosphatase, and catalase) were measured at 38, 62, and 137 days post-application, while bacterial and fungal community compositions were characterized via high-throughput sequencing of 16S rRNA and ITS2 genes. Our results demonstrated that the wa-60 treatment (30% wheat + 60% alfalfa) outperformed all the other formulations, delivering the highest yield (1.32 ± 0.08 kg/m^2^) and a 28.6% increase over the control (1.03 ± 0.06 kg/m^2^; *p* < 0.01), while maintaining a high total amino acid content (19.8 g/100 g). Soil analysis revealed that wa-60 notably enhanced urease activity (peaking at 85 µg/g/h at day 137) and alkaline phosphatase activity (322.55 µg/g/h), alongside significant increases in MBC and MBN. Microbiome profiling further demonstrated that wa-60 selectively increased the relative abundance of various taxa, particularly the bacterial phylum *Bacteroidota* and the fungal phylum Mortierellomycota. Correlation analyses indicated strong positive associations among these enriched taxa (primarily at the genus level), enhanced enzyme activities, and improved soil nutrient availability. Collectively, these findings establish that the wa-60 formulation, leveraging locally sourced alfalfa, represents a cost-effective and high-performance strategy for morel cultivation in the Qinghai Plateau, providing a microbial–ecological basis for optimizing ENB design and offering a practical pathway for recycling agricultural waste in edible mushroom production.

## Simple Summary

This study evaluated organic waste-based nutrient supplements for morel mushroom cultivation, focusing on yield, nutritional quality, and soil ecological responses. The wa-60 formulation increased yield by 28.6% while sustaining high amino acid levels, positioning it as a viable option for balancing productivity and fruit-body quality. Although wr-60 did not enhance yield, it substantially enriched soil bacterial diversity, indicating its potential for substrate amelioration and microecological restoration. Both treatments dynamically regulated soil organic matter, pH, macronutrients, microbial biomass, and enzyme activities, exhibiting a biphasic pattern of rapid initial changes followed by stabilization. Collectively, wa-60 emerged as a promising organic amendment strategy for morel production. Future research should incorporate field-scale trials and functional genomic approaches to clarify the causal roles of key microbial taxa in nutrient release and crop performance.

## 1. Introduction

*Morchella sextelata*, a prized edible and medicinal fungus, commands a high market price due to its culinary and therapeutic uses. It belongs to the taxonomic group phylum Ascomycota, order Pezizales, and family Morchellaceae and is highly regarded both for the exquisite taste of its fruiting body and for its diverse bioactive properties, including antioxidant, immunoregulatory, anti-inflammatory, anti-tumor, and anti-obesity activities [1,2]. *Morchella* spp. is rich in nutrients and bioactive compounds, which contribute to its nutritional and functional value. Owing to its high economic value, the artificial cultivation of *Morchella* spp. has expanded rapidly in China. Zhang et al. [3] isolated a water-soluble polysaccharide from *Morchella esculenta* and investigated its structural characteristics and biological activities. *Morchella* spp. are typically cultivated in greenhouse environments where air temperatures are maintained at approximately 10 °C, with winter temperatures kept above 3 °C to prevent frost damage. Soil temperature is generally controlled within the range of 6–15 °C, which is optimal for mycelial growth and primordium formation. Regarding soil conditions, Morchella does not exhibit strict substrate specificity.

Following the development and application of exogenous nutrient bag technology (ENB) technology, the artificial cultivation of *Morchella* spp. in China has undergone rapid expansion. This growth is clearly demonstrated by annual increases in both cultivation area and yield [4]. These bags are critical as they supply the growing mycelium with essential carbon and nitrogen sources that are typically scarce or unavailable in the soil. A core dilemma in large-scale morel cultivation is balancing the proven yield benefits of exogenous nutrient bags against their rising costs. To address this bottleneck, utilizing agricultural waste as an alternative substrate component has emerged as a promising solution. Studies indicate that mushroom residues are rich in nutrients essential for the growth and reproduction of various edible fungi, including crude protein, crude fat, crude fiber, polysaccharides, trace elements, vitamins, and minerals [5]. Wang et al. reported finding abundant *Morchella* spp. in fast-growing poplar forests, following the decomposition of residual substrates from the subsurface cultivation of oyster mushrooms and Coprinus comatus [6]. Zhang et al. demonstrated that the microbial community within the hyphal stage of *Morchella* spp. primarily engages in carbohydrate metabolism [7]. Zhang et al., in their study on wild *Morchella* spp., revealed that surface soils harbored significantly greater microbial diversity than deep soils, which was associated with higher organic matter content and a looser soil structure [8]. *Morchella* spp. are highly dependent on the soil microenvironment for hyphal growth and fruiting body formation, with soil microorganisms playing a central role. Studies indicate that both high soil bacterial community diversity and a high relative abundance of specific genera such as Pseudomonas can significantly promote morel development [9]. This was demonstrated in greenhouse studies of *Morchella sextelata*, where Pseudomonas was found to enhance both vegetative and reproductive growth [10]. More systematic studies are needed, particularly to elucidate how exogenous additives regulate the interaction networks within microbial communities. Recycled agricultural waste, including tomato substrate and coconut shells, acts as an effective soil amendment by improving physicochemical properties like pH, porosity, and nutrient levels, thereby enhancing crop yield and quality [11,12,13]. Crucially, the performance of these nutrient bag additives is closely linked to their regulation of the soil microbial environment. Therefore, utilizing waste materials such as straw should be encouraged to facilitate resource recycling.

As the Morchella cultivation industry continues to expand, the efficient utilization of cultivation nutrient bags and the development of localized, low-cost formulas tailored to different production regions have emerged as common technical challenges that urgently need to be addressed to enable sustainable development of the industry. Despite this, systematic studies on how exogenous additives from specific agricultural wastes (rapeseed straw, alfalfa, and oats) regulate the interaction networks within soil microbial communities are lacking. Crops such as oilseed rape, alfalfa and oats are widely cultivated in China, yielding enormous volumes of agricultural by-products. However, much of the resulting straw and processing residues have not been put to high-value use. This not only leads to the underutilization of biomass resources, but also poses environmental risks, including open-field straw burning. Using these residues as substrate components may provide an alternative pathway for biomass recycling Addressing these scientific questions, this study aims to analyze the soil microbial interaction network following amendment with rapeseed straw, alfalfa, and oats by monitoring soil indicators, including pH, total and available nutrients, enzyme activities, and microbial biomass, as well as morel yield and amino acid composition. This study aims to investigate the shifts in soil microbial community structure and their associations with key environmental variables after amendment with rapeseed straw, alfalfa, and oats. The results are expected to provide new pathways for improving Morchella culture substrates through the regulation of microbial communities, thereby promoting the transformation and upgrading of agricultural resources.

## 2. Materials and Methods

### 2.1. Study Site and Soil Characteristics

The field experiment was conducted at a farmer’s greenhouse in Angla Village, Jianzha County, Huangnan Prefecture, Qinghai Province (35°53′ N, 102°3′ E). Before the nutrient bag amendment experiment, the protocol included lime (CaO, 750 kg/ha), tillage at ~25 cm, and a 7-day post-solarization wait before inoculation. The site experiences a continental plateau valley climate, while the greenhouse temperature is maintained at 10–25 °C throughout the year. The soil was classified as a sandy loam. Key soil characteristics included a pH of 8.65 and an organic matter content of 14.95 g/kg. Total nitrogen (TN), total phosphorus (TP), and total potassium (TK) were 0.87 g/kg, 1.07 g/kg, and 20.62 g/kg, respectively. The available phosphorus and potassium levels were 51.56 mg/kg and 489.9 mg/kg.

### 2.2. Experimental Materials

*Morchella sextelata* strain QJ-2 (the mother culture is maintained on potato dextrose agar (PDA) slant tubes at 4 °C and periodically activated; for propagation, a small amount of mycelium is carefully picked from the tube with an inoculating needle, placed onto a PDA plate, and incubated at 22 °C for 3–5 days for activation; the growing mycelium is then transferred to a fresh plate for propagation) was used by the research team at Qinghai University’s State Key Laboratory of Plateau Ecology and Agriculture. To leverage local resources, we used regionally sourced rapeseed straw (Qingza 7), alfalfa, and oats (Qingyan 2) as amendments in the nutrient bags. While wheat and corn cob are commonly used in morel cultivation, we opted for these locally available materials. Each bag measured 15 × 10 cm and had a wet weight of 300 g.

### 2.3. Experimental Design

We employed a randomized complete block design with three blocks: CK (conventional formulation control), wco-30 (30% organic fertilizer), wca-30 (30% alfalfa), wcg-30 (30% oats), wcag-15 (15% alfalfa–oats mix), wr-60 (60% rapeseed straw), wo-60 (60% organic fertilizer), wa-60 (60% alfalfa), wg-60 (60% oats), and wag-30 (30% alfalfa–oats mix). The experiment was arranged in a randomized complete block design with three blocks. Each block contained all ten treatments, and the individual 6 m^2^ plot within each block for a given treatment constituted one biological replicate (*n* = 3 per treatment). Within each plot, nutrient bags were placed in 3 rows with a 25 cm spacing, with 8 bags per row at 20 cm intervals along the rows. Spawn of *M. sextelata* strain QJ-2 was inoculated on 12 October 2024, and nutrient bags were placed on 26 October, followed by covering with black plastic film. Exogenous nutrient bags were placed once and not replenished during the growing period. All other field management practices were kept consistent across treatments. The specific formulation treatments are detailed in Table 1.

### 2.4. Soil Sampling and Sample Preparation

Sampling dates were chosen to correspond to key stages of the Morchella cultivation cycle: Soil samples were collected from the 0–20 cm layer on four dates corresponding to key stages of the Morchella cultivation cycle: 12 October 2024 (baseline, before inoculation, 0 day); 3 December 2024 (38 days after ENB placement, mycelial colonization); 27 December 2024 (62 days after ENB placement, primordium initiation); and 12 March 2025 (137 days after ENB placement, fruiting body maturation and harvest). For each 6 m^2^ plot, three soil cores were randomly collected from the 0–20 cm layer using a stainless-steel auger and pooled to form a single composite sample, which was then homogenized and transferred to a sterilized 50 mL centrifuge tube. In total, three composite samples were obtained per treatment (one from each of the three replicate plots), representing three biological replicates (*n* = 3). The samples collected at the final sampling time were immediately placed in foam boxes with dry ice (approximately −80 °C) and transported to Guangzhou Jidiao Technology Co., Ltd. for soil microbial diversity analysis. Soil samples were collected from the 0–20 cm depth layer after removal of the surface litter and visible plant residues. Following indoor air-drying, the samples were ground and sieved through a 2 mm sieve for analysis of soil physicochemical properties.

### 2.5. Determination of Soil Physicochemical Properties, Microbial Biomass, and Enzyme Activities

We quantified the key soil chemical properties using established procedures: soil organic matter was determined by the potassium dichromate heating method [14], and total nitrogen was measured using a flow analyzer (model: Auto Analyzer 3-AA3, manufactured by SEAL Analytical, Norderstedt, Germany) after digestion with sulfuric acid and an accelerator [15]. Total phosphorus was digested with a mixture of sulfuric acid and perchloric acid, followed by molybdenum–antimony colorimetric determination [14]. Total potassium was determined with a flame atomic absorption spectrometer (PinAAciie 900F, PerkinElmer, Waltham, MA, USA) following sodium hydroxide fusion [14]. Alkali-hydrolyzable nitrogen was measured via the alkali diffusion method [16]. Available phosphorus was extracted with sodium bicarbonate and quantified by molybdenum–antimony colorimetry using a UV-Vis spectrophotometer (UV-2450, Shimadzu, Kyoto, Japan) [17]. Available potassium was extracted with ammonium acetate and determined by flame atomic absorption spectrometry (PinAAciie 900F, PerkinElmer) [18]. The concentrations of nitrate and ammonium nitrogen were determined using a flow injection analyzer [19]. Microbial biomass carbon (MBC) was analyzed with a TOC analyzer (TOC-L, Shimadzu, Kyoto, Japan) [20]. Microbial biomass nitrogen (MBN) and phosphorus (MBP) were determined by chloroform fumigation followed by flow injection analysis [21]. Enzyme activities were assessed as follows: urease by indophenol blue colorimetry [22], alkaline phosphatase using p-nitrophenyl phosphate disodium colorimetry [23], protease activity by a spectrophotometric method, and catalase activity via potassium permanganate titration [23].

We analyzed soil microbial data on the Gidio (Guangzhou, China) cloud platform (https://www.omicsmart.com/#/) (accessed on 15 August 2026). The experimental data were initially compiled in Microsoft Excel 2016, after which a one-way ANOVA was run on SPSS Statistics 24. Tukey’s method served for multiple comparisons, and the significance of differences was evaluated at the *p* < 0.05 level.

### 2.6. DNA Extraction, PCR Amplification, and Amplicon Sequencing

Soil samples were transported to the laboratory on dry ice within 6 h of collection and stored at −80 °C until DNA extraction. Total DNA was extracted within two weeks of collection from frozen samples. Total soil DNA was extracted from 0.5 g of soil per sample using the HiPure Soil DNA Kit (D3142; Guangzhou Magen Biotech Co., Ltd., Guangzhou, China) according to the manufacturer’s protocol. After extraction, DNA integrity was evaluated by agarose gel electrophoresis, and DNA purity and concentration were measured using the NanoDrop 2000c (Thermo Scientific, Wilmington, DE, USA). The V3 + V4 region of the bacterial 16S rRNA gene was amplified with the following primer sets: 341F (5′-CCTACGGGNGGCWGCAG-3′) and 806R (5′-GGACTACHVGGGTATCTAAT-3′). The ITS2 fragment of fungi was amplified using the forward primer ITS3_KYO2 (5′-GATGAAGAACGYAGYRAA-3′) and the reverse primer ITS4 (5′-TCCTCCGCTTATTGATATGC-3′). The same thermal cycling conditions were applied to both 16S and ITS amplifications. PCR amplification was performed in a total volume of 50 μL, which contained 5 μL of 1× Pfu PCR buffer, 10 μL of dNTP mixture (2.5 mM each), 1.5 μL each of forward and reverse primers (10 μM), 0.2 μL of Pfu DNA polymerase, 50 ng of template DNA, and ddH_2_O added to make the quantity up to 50 μL. PCR conditions included: 5 min pre-degeneration at 95 °C before cycling and 30 cycles of 1 min denaturation at 95 °C, 1 min annealing at 60 °C, and 1 min extension at 72 °C, followed by a final 7 min extension at 72 °C.

### 2.7. Bioinformatics Analysis

In this study, paired-end sequencing (PE250) was carried out on an Illumina NovaSeq 6000 platform (Illumina, San Diego, CA, USA), using the Illumina DNA Prep Kit (cat. no. 20060059) and the NovaSeq 6000 S2 Reagent Kit (cat. no. 20042038) for library construction and sequencing, respectively. For bacterial sequences, taxonomic assignment was performed against the SILVA 138.1 16S rRNA database; for fungal sequences, it was performed against the UNITE ITS database. All other processing steps (quality filtering, DADA2 denoising, and chimera removal) were applied independently to each dataset as recommended for each marker gene. Raw FASTQ data were demultiplexed according to sample barcode information using QIIME 2 version. Quality control and filtering were then carried out with criteria including: average Phred score > 20, no ambiguous bases, homopolymer runs < 8, no mismatches in primers, and read length > 250 bp. After filtering, paired reads were merged, and chimeras were removed using the DADA2 built-in pipeline. ASVs were inferred from the clean sequences using DADA2. Singletons (ASVs with only one read) were discarded, and the remaining data were normalized to the lowest read count across all samples. Taxonomic assignment of ASVs was performed against the SILVA database. Subsequently, based on ASV abundance information, analyses of species diversity, Tukey HSD, material correlations, and functional predictions (using PICRUSt2 version 2.2.0 and FUNGuild version 1.0) were conducted. Finally, microbial community structures and functional characteristics were obtained through principal coordinate analysis (PCoA), Functional Annotation of Prokaryotic Taxa (FAPROTAX), redundancy analysis (RDA), correlation heatmaps, and differential abundance analysis.

The entire workflow was mainly implemented using QIIME2 version 2024 with the DADA2 plugin. The alpha diversity indices of the bacterial and fungal communities, including the Chao1 index, Shannon index, Sobs index and Simpson index, were calculated. Differences in these alpha diversity indices and soil porosities between different soil depths were analyzed using the Student’s *t*-test, while differences in the soil between different times and treatments were analyzed using Tukey’s HSD test. Variations in the microbial community compositions of the soils from different years and at different soil depths were evaluated via principal coordinate analysis (PCoA) based on the Bray–Curtis distance at the ASV level, and permutational multivariate analysis of variance (PERMANOVA) was applied to test the significance of group differences. Additionally, redundancy analysis (RDA) was performed to explore the relationships between microbial community structure and key soil environmental variables. In addition, we annotated the functions of bacteria using the Functional Annotation of Prokaryotic Taxa (FAPROTAX) database, focusing on microbial abundance changes in microbial processes related to nitrogen metabolism.

## 3. Results

### 3.1. Effects of ENB Formulations on Morel Yield and Amino Acid Composition

**Figure 1:**
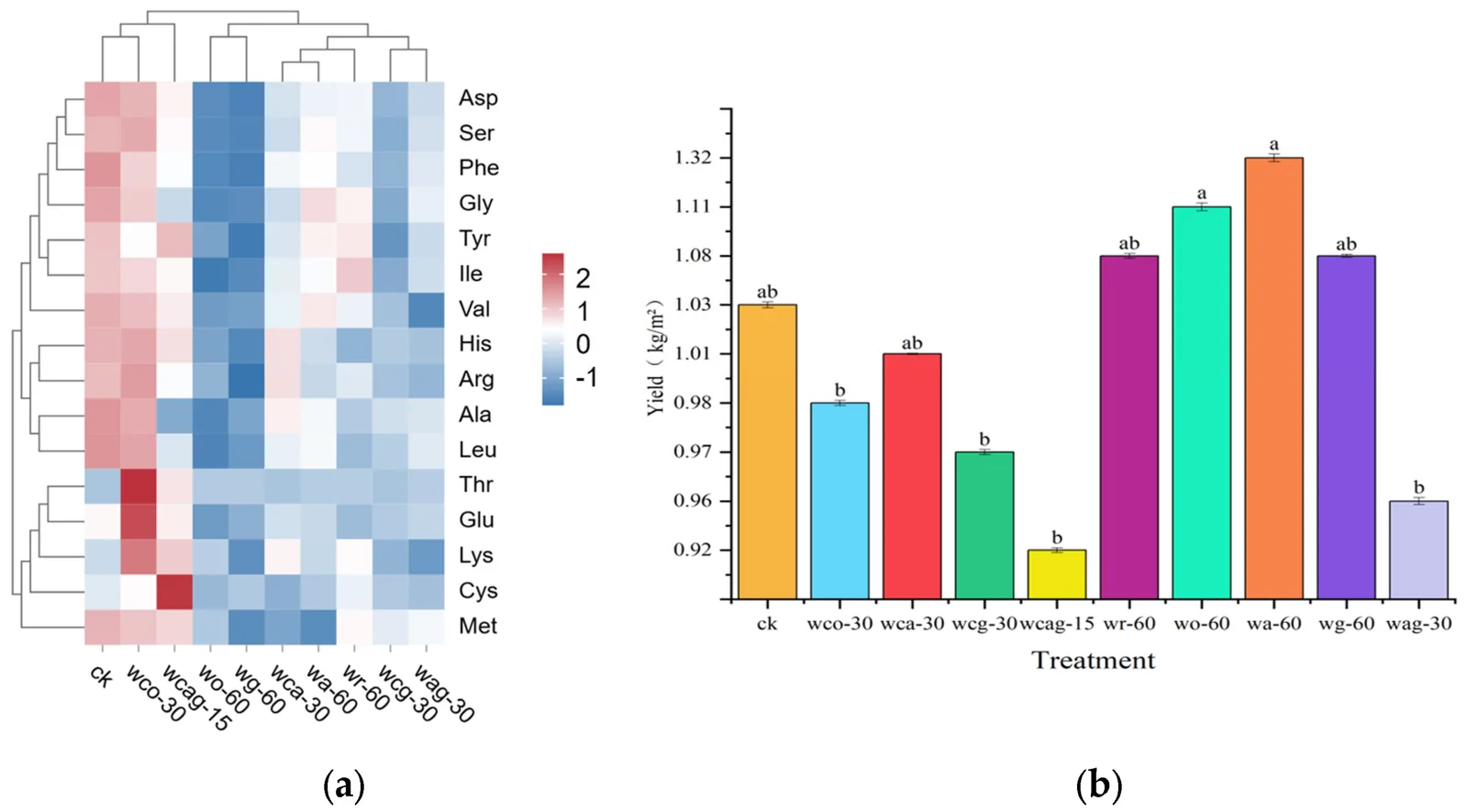
Amino acid profiles and yield of Morchella fruiting bodies cultivated with different organic amendment formulations. (**a**) Heatmap showing relative abundance of 16 amino acids across treatments, normalized by Z-score. Hierarchical clustering was applied to both rows (amino acids) and columns (treatments). (**b**) Yield (kg·m^−2^) of each treatment, with error bars representing SDs (*n* = 3). Different lowercase letters above bars indicate significant differences between treatments (one-way ANOVA followed by Tukey’s HSD, *p* < 0.05).

We observed a clear increase in essential amino acids like threonine and lysine under several treatments (e.g., wco-30), whereas aspartic acid and leucine content decreased consistently across amendments (Figure 1). Dry weight remained relatively stable across the experiment, and most treatments did not differ substantially from the control. While only the wa-60 treatment achieved the highest yield, the wr-60, wo-60, and wg-60 treatments also produced relatively high yields. Most other formulations resulted in lower production than the control, indicating that the yield did not differ significantly from that of CK. The wa-60 formulation yielded 1.32 ± 0.08 kg/m^2^, a 28.6% increase over the CK group (1.03 ± 0.06 kg/m^2^; *p* < 0.05). The yield from wr-60 was 1.08 ± 0.05 kg/m^2^, which was significantly lower than that of wa-60 (*p* < 0.05). The significant variations in some of the amino acid composition and yield indicators between treatments suggest that ingredient formulation and processing play a decisive role in determining morel quality and yield. The superior performance of wa-60 may be attributed to the favorable carbon-to-nitrogen (C/N) ratio and lignin content of alfalfa, which likely provided a balanced nutrient supply for mycelial growth and primordium formation—a hypothesis that is further explored in the Discussion (Section 4.1).

### 3.2. Effects of ENB Formulations on Soil Physicochemical Properties

Table 2 depicts the temporal dynamics of eight key soil physicochemical properties, showing their evolution over 38, 62, and 137 days across the different treatments. Overall, most indicators exhibited a trend of initial increase followed by a subsequent decrease: values peaked at day 62, followed by a moderate decline by day 137. Despite this, levels generally remained elevated compared to the measurements at day 38. These patterns demonstrate a distinct time-dependent effect of the management practices. Concurrently, the magnitude of differences between treatments increased progressively over time: at day 38, treatment effects were relatively modest, whereas by day 137, the separation between treatments became most pronounced. The soil background remained stable, with pH never straying from the 8.2–8.9 range.

### 3.3. Effects of Different Formulations on Soil Enzyme Activities and Microbial Biomass

Table 3 documents temporal shifts in microbial biomass and key enzymes. While MBC generally increased from 38 to 137 days for most treatments, the temporal patterns were not uniformly upward; certain treatments (CK, wr-60, and wg-60) displayed a dip-then-rise trajectory. The treatment-specific responses nonetheless support that nutrient amendments enhanced microbial carbon utilization and biomass growth. For most treatments, microbial biomass nitrogen (MBN) was consistently higher at day 137 than at day 38, indicating a strengthened microbial capacity for nitrogen immobilization over time. A distinct variation was observed in MBP enhancement across most treatments. Notably, the wag-30 treatment significantly increased MBP levels, proving more effective than both the w-o and CK groups. The MBC/MBN ratio, which reflects the relative dominance of bacterial vs. fungal biomass in the microbial community, ranged from 6.8 to 12.5 across treatments at day 137. Notably, the wa-60 treatment exhibited a ratio of 9.3, close to the theoretical threshold for balanced bacterial–fungal communities, whereas wr-60 showed a higher ratio (11.8), suggesting a relatively greater fungal contribution in this treatment. The MBC/MBP ratio remained relatively stable (ranging from 18.5 to 24.6) across treatments, indicating no major shifts in phosphorus allocation per unit of microbial biomass.

At day 38, the wcg-30 and wo-60 treatments already exhibited significantly higher UR activity. By day 137, the wcag-15 group reached a peak activity of 85 µg/g/h, representing a 37.1% increase over the control and indicating a sustained enhancement of organic nitrogen transformation. The significant variation in UR activity between treatments collectively demonstrates that the nutrient bags enhance urease function, thereby accelerating the conversion of organic nitrogen to ammonium nitrogen. The sustained rise in Acid Phosphatase Activity (APA) was evidenced by increases such as wr-60’s increase from 150 to 300 μg g^−1^ h^−1^ by day 137. This correlation points to microbial growth, especially of phosphobacteria, as the driver of enhanced enzyme production to mobilize organic phosphorus. In contrast, catalase (CAT) activity showed a relatively narrow range of variation across treatments, with no significant difference between groups (e.g., CK and wco-30). This limited variation implies that catalase is less responsive to these environmental changes, or that the treatments induced a similar degree of oxidative stress. Notably, changes in urease and alkaline phosphatase activities were positively correlated with MBC and MBN across treatments (urease vs. MBN: r = 0.68, *p* < 0.05; APA vs. MBC: r = 0.71, *p* < 0.05), suggesting that the observed increases in enzyme activities were at least partly driven by the expansion of microbial biomass.

### 3.4. Effects of Different Formulations on the Diversity of Soil Microbial Communities

#### 3.4.1. Alpha Diversity of Soil Bacterial and Fungal Communities

Analysis using the Sobs, Shannon, Simpson, and Chao1 indices (Table 4) demonstrates that the different nutrient bag treatments significantly altered the alpha diversity of the soil microbial communities.

The α-diversity indices of soil bacterial communities were significantly affected by different treatments. The Sobs and Chao1 indices, reflecting species richness, shared similar variation trends. The wr-60 treatment exhibited the highest Sobs and Chao1 values, followed by wca-30, wag-30 and wcag-15. In contrast, wa-60 significantly reduced bacterial species richness, with the minimum Sobs value among all groups. The Shannon index, representing community diversity, reached the maximum under wr-60 treatment, while wa-60 obtained the lowest Shannon index. No significant differences in Simpson index were observed across all treatments, indicating that all managements hardly altered bacterial community dominance. The observed differences in Shannon diversity between wco-30 (6.18) and wa-60 (3.97) for bacteria, while statistically significant (*p* < 0.05), also represent a biologically meaningful difference in community diversity—equivalent to a fold change of 1.56 in species richness. This magnitude suggests that wco-30 not only increased statistical diversity but also substantially expanded the functional potential of the bacterial community. Collectively, wr-60, wca-30 and wag-30 effectively improved soil bacterial richness and diversity, whereas wa-60 exerted adverse effects on bacterial α-diversity.

Among the soil fungal Sobs and Chao indices, the wco-30 treatment was most notable, recording the highest values (544.33), which were significantly greater than those of most of the alternatives, demonstrating its superior capacity for expanding the microbial species pool. Regarding community diversity and evenness (Shannon and Simpson indices), the wco-30, ck, wca-30, wcg-30, and wr-60 treatments showed relatively high Shannon and Simpson index values. Their high index values (Shannon: 5.78–6.18; Simpson: 0.95–0.96) showed no significant intra-group differences, indicating more complex and stable community structures.

#### 3.4.2. Beta Diversity of Soil Bacterial and Fungal Communities

**Figure 2:**
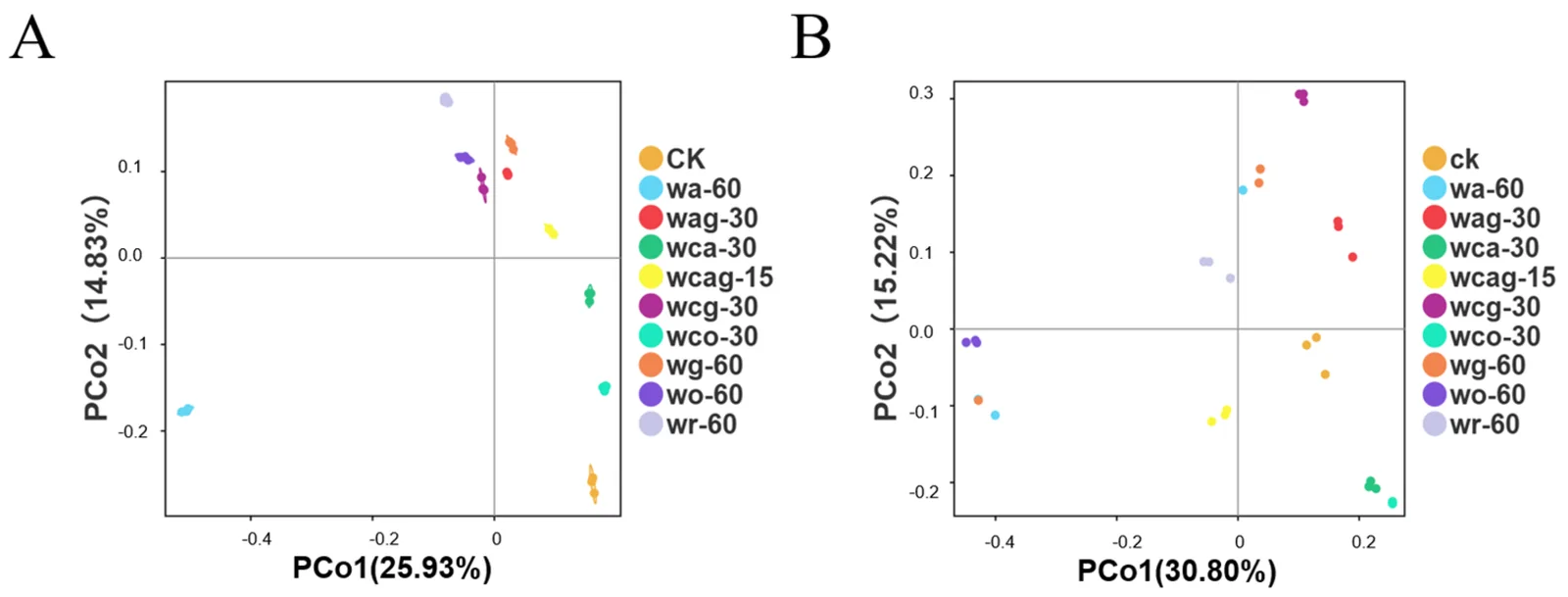
Principal coordinate analysis (PCoA) of soil microbial communities under different exogenous nutrient bag (ENB) formulations based on Bray-Curtis dissimilarities. (**A**) Bacterial community. (**B**) Fungal community.

The PCoA of bacterial communities (Figure 2A) showed that treatments were a key factor driving the separation, and a similar separation pattern was observed for fungal communities (Figure 2B), also with treatments as the primary structuring factor. Relative to the control, which occupied a unique position in the ordination, the wa-60 and wca-30 treatments each resulted in a distinctive restructuring of the soil bacterial communities. In contrast, the five treatments—wg-60, wag-30, wo-60, wr-60, and wcg-30—clustered closely together, indicating a comparable ecological impact on the microbial community. This pattern was captured by a PCoA where PC1 and PC2 explained 25.93% and 14.83% of the variation. For fungal communities, the coordinates explained 30.80% and 15.22% of the variation. The separation of the wa-60 group along PC1 in the fungal PCoA (Figure 1b) was closely associated with the higher soil organic matter (SOM) and nitrate nitrogen (NO_3_^−^-N) levels observed in this treatment (Table 3). Similarly, the distinct positioning of the wco-30 group in the bacterial PCoA (Figure 1a) coincided with its having the highest bacterial richness (Sobs index = 544.3). These patterns suggest that the beta-diversity shifts observed here reflect the underlying variation in soil chemical properties induced by the different ENB formulations.

#### 3.4.3. Composition of Soil Bacterial and Fungal Communities Under Different ENB Formulations

##### Soil Bacterial Community Composition

**Figure 3:**
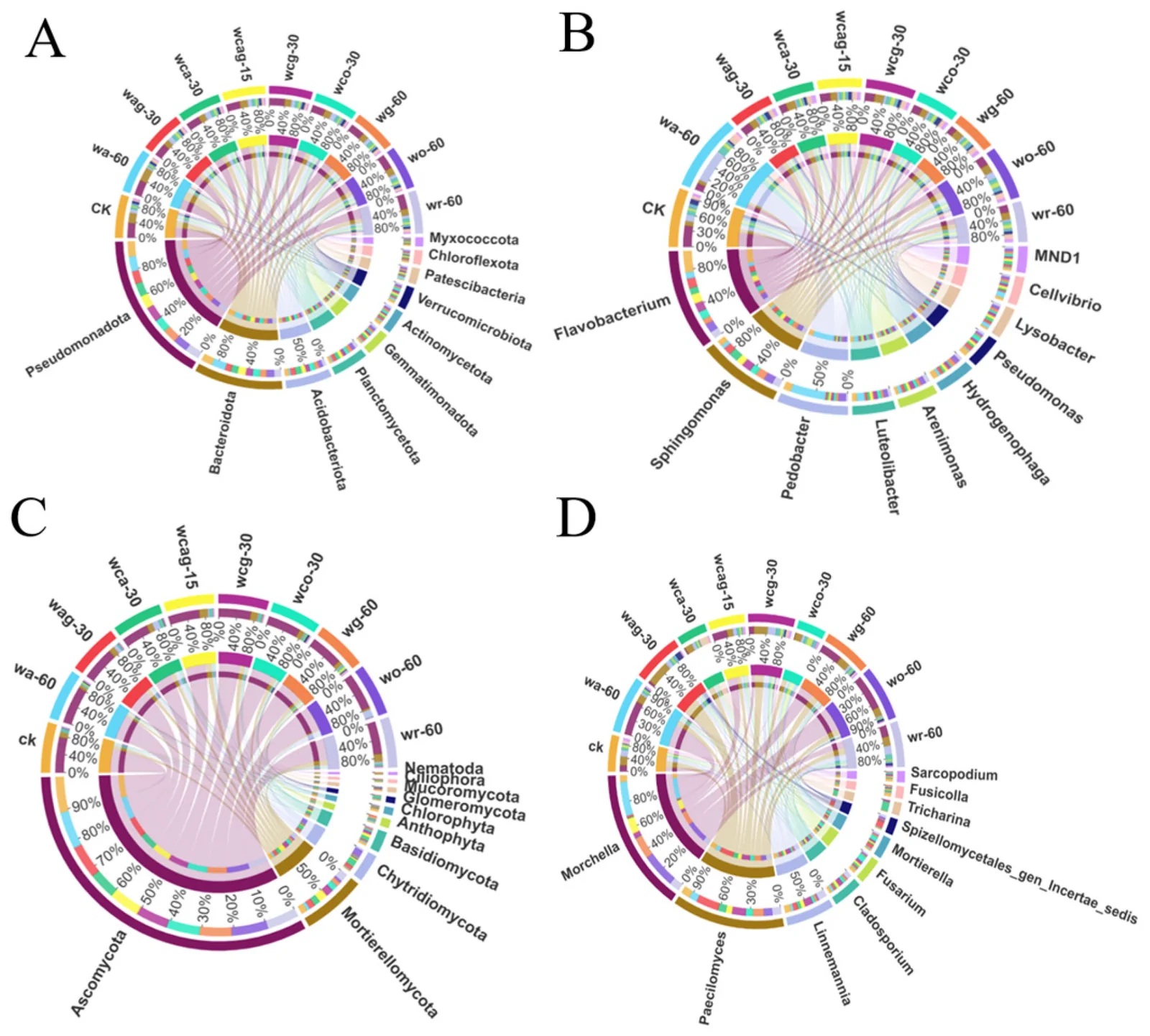
Relative abundance of soil communities at the phylum (**A**,**C**) and genus (**B**,**D**) levels across different ENB treatments. Taxonomic groups with a mean relative abundance < 1% across all samples were consolidated into the category “Others”.

As shown in Figure 3A, the relative abundance of *Pseudomonadota* exceeded 30% in all treatments, while *Bacteroidota* generally accounted for more than 10% (particularly over 20% in the wa-60 treatment). *Planctomycetota* exceeded 3% in all groups but were relatively low in the CK control. Other phyla, including *Acidobacteriota*, *Actinomycetota*, and *Gemmatimonadota*, also exhibited variations between treatments. Figure 3B reveals that *Flavobacterium*, *Sphingomonas*, MND1, and *Pseudomonas* were the core dominant genera shared across all samples. The treatments exerted significant selective regulation of community structure; wa-60 and wag-30 markedly reduced the relative abundance of *Flavobacterium* from 9.19% in CK to 2.81% and 4.17%, respectively. In contrast, other genera, such as MND1 and *Pseudomonas*, showed only minor absolute fluctuations (MND1 ranging from 0.85% to 1.13% and *Pseudomonas* ranging from 0.63% to 1.67% across treatments), with no clear directional trends. These differential genera are mostly associated with key ecological functions, such as environmental remediation, nitrogen cycling, and biocontrol, suggesting that different treatments were associated with distinct taxonomic shifts in specific genera, contributing to alterations in community composition.

##### Soil Fungal Community Composition

Figure 3C illustrates differences in the relative proportions of major fungal phyla. The *Ascomycota* dominated all groups (>70% relative abundance), reaching nearly 80% in wa-60 and wo-60, demonstrating their remarkable ecological success. Among fungal phyla, the most pronounced treatment effects were observed for *Chytridiomycota*, which increased from 0.74% in ck to 15.24% in wo-60 and 13.73% in wca-30 (~19- and 17-fold). *Mortierellomycota* was highest in wca-15 (18.83%) and wr-60 (17.30%), while *Ascomycota* peaked in wa-60 (78.58%) but dropped to 56.40% in wca-30. *Basidiomycota* was enriched by wca-30 (7.86%) and suppressed by wo-60 (0.86%). *Glomeromycota* peaked in wco-30 (3.58%, ~5-fold). In Figure 3D, the fungal genus-level responses were treatment-specific: wcg-60/wg-60 enriched *Morchella* (~46%), wca-30 boosted *Tricharina* (0.02–11.57%), and wag-30 raised *Paecilomyces* (31%) and *Spizellomyces* (7.9%), while *Fusicolla* and *Sarcopodium* declined across treatments (especially <0.25% in wg-60), indicating that treatments reshape community structure through differential stimulation or suppression of key genera.

#### 3.4.4. Biomarkers and Indicator Taxa

**Figure 4:**
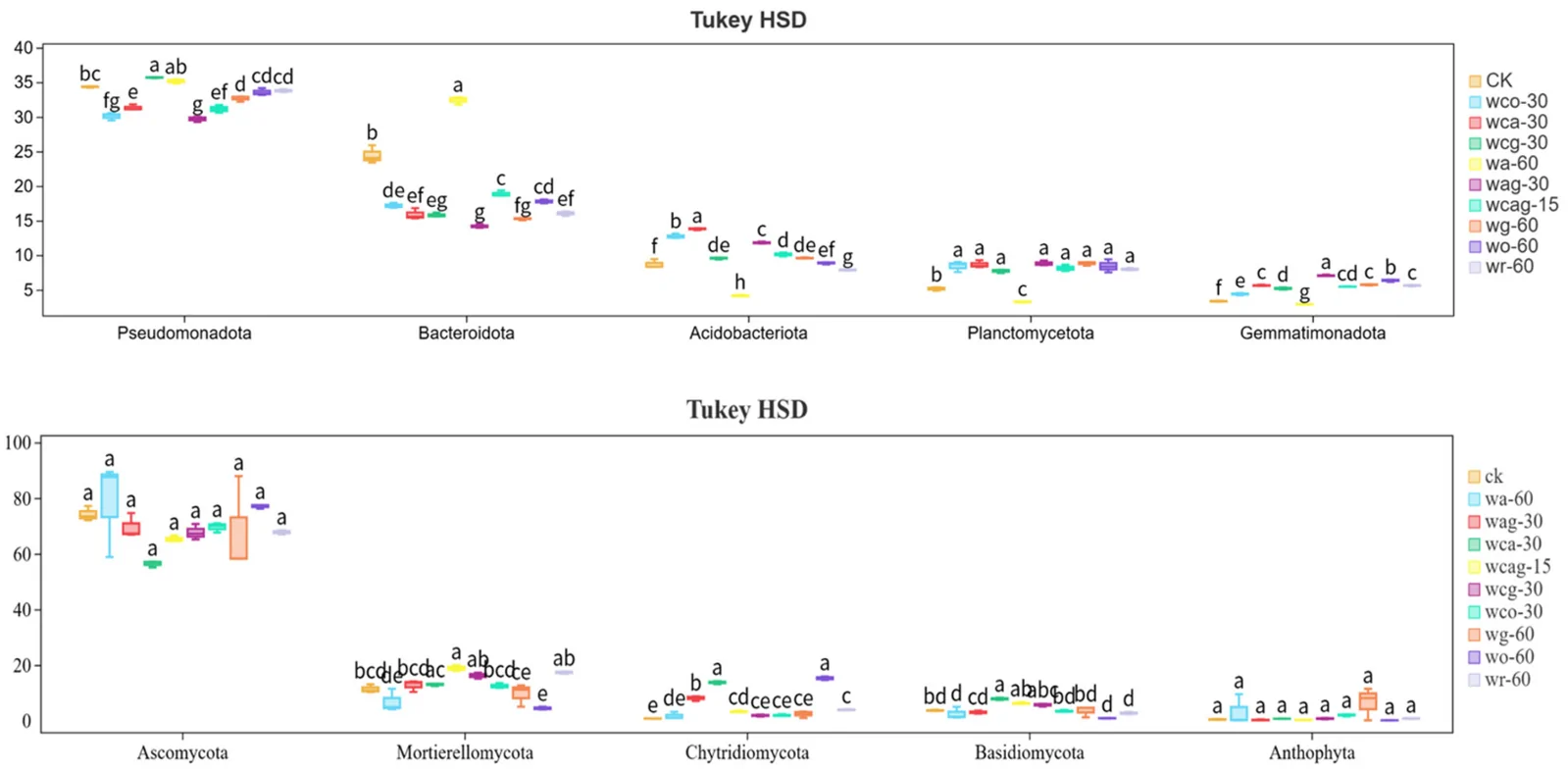
Composition of soil indicator species under different formulations. Note: This letter-based labeling is only valid for comparisons among treatments within the same phylum; letters across different phyla are not comparable.

Tukey’s HSD post hoc test revealed significant taxon-specific variations in core microbial groups between the treatments (Figure 4). The relative abundances of five microbial taxa (the genus Pseudomonas and the phyla *Bacteroidota*, *Acidobacteriota*, *Planctomycetota*, and *Gemmatimonadota*) across treatments were largely stable, with values ranging narrowly between 28.5% and 30.1%. No clear treatment-dependent enrichment or depletion was observed for these taxa.

At the taxonomic level, *Glomeromycota* were uniquely enriched in the wco-30 treatment, with a relative abundance of 0.35. *Chlorophyta* reached its peak abundance (0.30) in the ck group but was generally suppressed in treated plots. Furthermore, Tukey’s HSD test revealed no significant differences in *Mortierellomycota* or Basidiomycota abundances between the treatments, yet their systematic variations across treatments suggested a consistent modulation by micro-environmental conditions. These taxon-specific responses form a functional bridge connecting agricultural management with soil ecological processes.

#### 3.4.5. Relationships Between Soil Microbial Communities and Soil Properties

##### Linking Soil Fungal Communities to Chemical Properties

**Figure 5:**
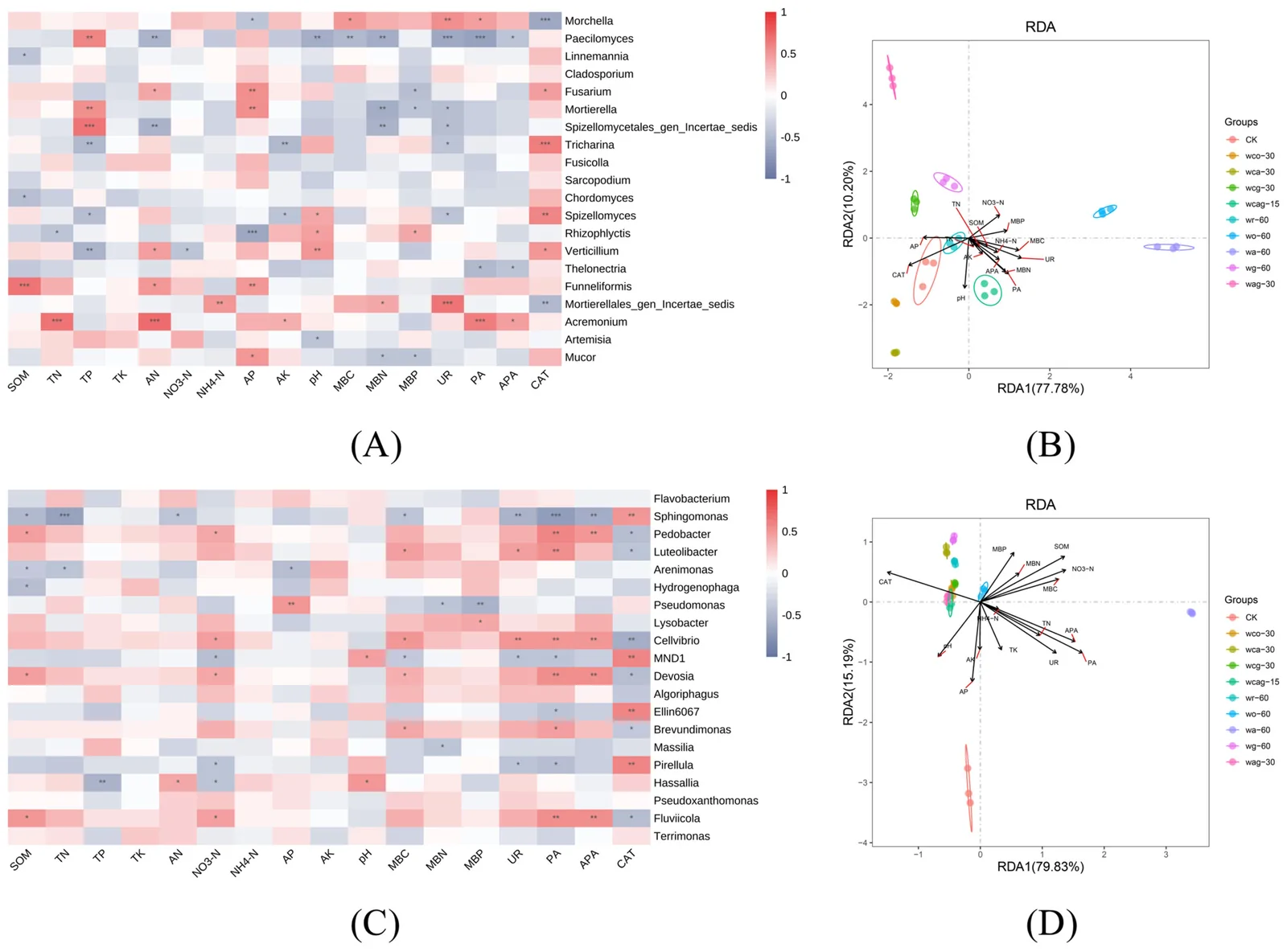
RDA ordination of soil fungal (**A**,**B**) and soil bacteria (**C**,**D**) communities with environmental variables. SOM, soil organic matter; TN, total N; TP, total P; TK, total K; AN, available N; NO_3_^−^-N, nitrate-N; NH_4_^+^-N, ammonium-N; AP, available P; AK, available K; MBC/MBN/MBP, microbial biomass C/N/P; UR, urease; PA, protease; APA, acid phosphatase; CAT, catalase. Linking Soil Bacterial to Chemical Properties Asterisks (correlation heatmap): In the genus–soil environmental factor correlation heatmap, asterisks indicate the significance level of the correlation coefficient: * for *p* < 0.05 (significant), ** for *p* < 0.01 (highly significant), and *** for *p* < 0.001 (extremely significant). Red shading represents positive correlations, blue shading represents negative correlations, and color intensity corresponds to the magnitude of the correlation coefficient.

The correlation heatmap in Figure 5A reveals the complex relationships between the soil fungal community and key environmental factors. Our analysis revealed distinct correlation patterns between different fungal taxa and environmental factors, resulting in clear ecological niche differentiation. The relative abundance of *Morchella* growth was closely linked to soil nitrogen cycling and microbial activity, as evidenced by its highly significant positive correlations with both urease and microbial biomass carbon. Conversely, Morchella was inversely related to catalase activity, suggesting that the oxidative stress response may constrain its growth. *Fusarium* and *Acremonium* exhibited highly specific nutrient correlations, the former showing a strong positive link to available phosphorus and the latter demonstrating a similar relationship with alkali-hydrolyzable nitrogen. As for additional relationships, *Spizellomycetales* showed a significant positive correlation with total phosphorus (TP).

Figure 5B shows a clear clustering pattern across the groups. The wag-30 group stands apart from the others in the upper left section. The ck, wco-30, and wca-30 groups are clustered together in the bottom left area, while the wcag-15 samples were located in the lower portion of the ordination plot. The wo-60 and wa-60 groups cluster together on the right side of the plot. Factors such as pH, CAT, AP, and NO_3_^−^-N show long arrows pointing toward distinct groups, suggesting that they were associated with community variation. NO_3_^−^-N and MBP showed stronger associations with groups such as wo-60. Overall, RDA1 and RDA2 together explained approximately 88% of the total variation (77.78% and 10.20%, respectively), indicating that treatment regime and environmental factors jointly contributed to the observed community separation.

The correlation analysis in Bacterial 5(C) reveals specific associations between microbial genera and soil factors. The relative abundance of *Pseudomonas* showed a strong positive correlation with available phosphorus, pointing to a strong dependence on this readily available nutrient source. Enzyme activities were associated with distinct regulatory patterns among bacterial genera: both *Flavobacterium* and *Pseudomonas* showed significant positive correlations with protease activity, while *Sphingomonas* exhibited a significant negative correlation with protease activity. These taxa likely are collectively positively correlated with the relative abundance of organic nitrogen and phosphorus in the soil. Conversely, *Cellvibrio* abundance showed a strong negative correlation with catalase activity, suggesting it may avoid or suppress this antioxidant process—reflecting a divergent ecological strategy from other genera.

Figure 5D shows that RDA1 and RDA2 together explain 95.02% of the total variation, clearly revealing the community differentiation patterns among samples from different treatment groups: the ck samples are positioned in the lower middle part of the RDA ordination, separating significantly from other treatment groups; most treatment groups, including wco-30 and wca-30, cluster near the origin, with relatively similar community structures; the wa-60 group is located alone at the far right end, showing a significant positive correlation with factors such as SOM, NO_3_^−^-N, and MBC. Overall, the results indicate that different treatment regimes were associated with distinct changes in community structures by regulating key environmental factors.

#### 3.4.6. Predicted Nitrogen-Cycling Functions and Fungal Ecological Guilds

**Figure 6:**
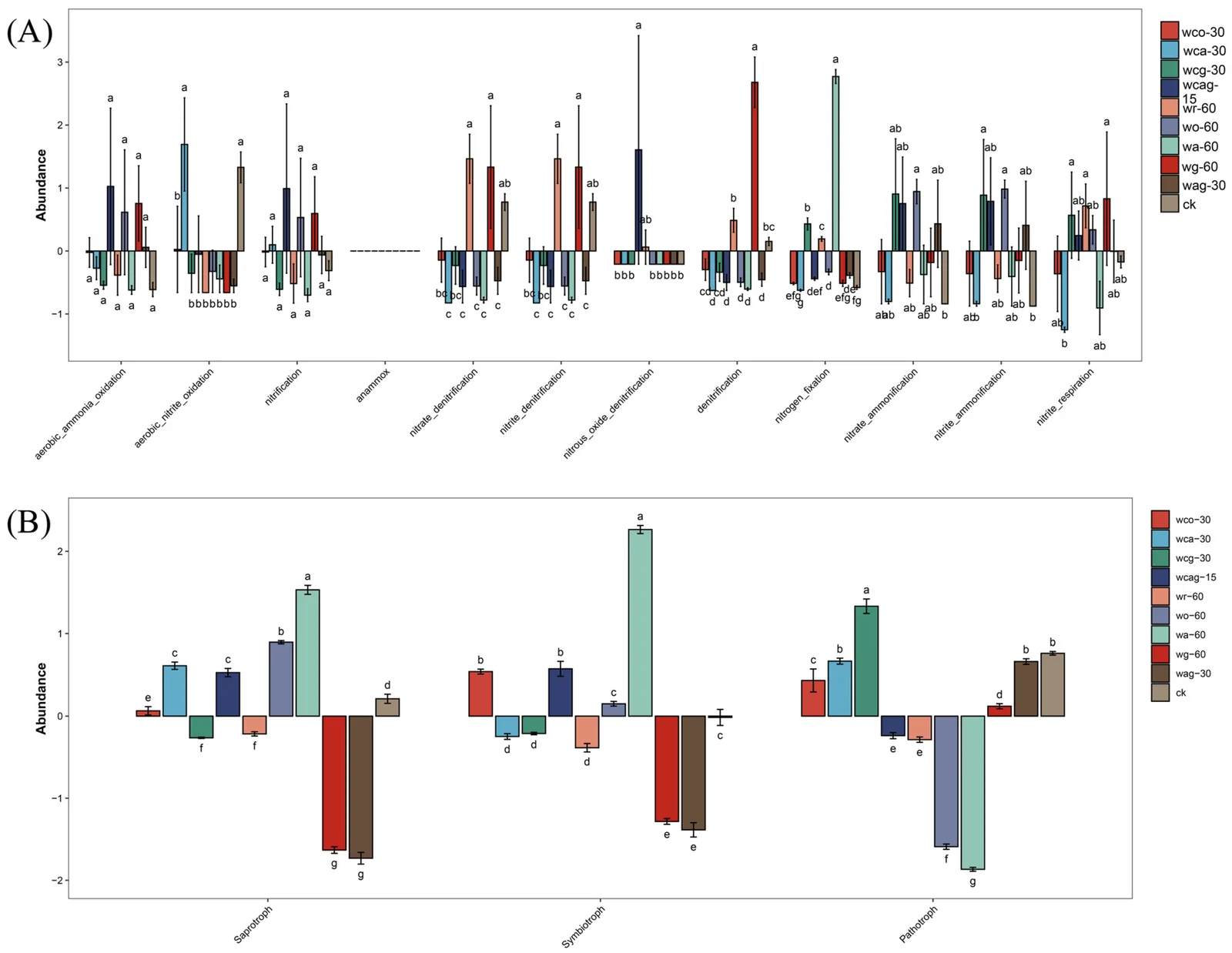
Predictive analysis of FAPROTAX functions (**A**) and FUNGuild functions (**B**) in different formulations. Note: Letter comparisons are only valid among treatments within the same functional pathway; letters across different functional groups are not comparable.

FAPROTAX functional annotation analysis (Figure 6) revealed a total of 12 functional groups within the soil bacterial communities of the resistant varieties, with significant differences observed across treatments. Pairwise statistical comparisons (Kruskal–Wallis tests with FDR correction) revealed no significant differences between treatments for nitrogen fixation, anaerobic ammonia oxidation, or nitrate respiration (*p* > 0.05). In contrast, the nitrification, denitrification, and ammonification pathways exhibited relatively higher responsiveness to the treatments. Overall, most predicted functional categories exhibited higher relative abundances in treatment groups than in the control, with the ammonification pathway showing a slightly increasing trend. FUNGuild functional prediction additionally uncovered that The wcg-30 treatment significantly enriched pathotrophic fungi, whereas wa-60 primarily enriched symbiotrophic and saprotrophic fungi.

## 4. Discussion

### 4.1. Yield and Amino Acid Responses to Different ENB Formulations

Research by Chen Yuanwen et al. [24] indicates that ENBs are critical for fruiting body formation in Morchella: their absence nearly prevents yield, whereas their application significantly enhances both formation and productivity. The practice of agricultural straw turnover enhances nitrogen fertilizer utilization and activates the soil carbon pool. *Morchella* spp. contribute to this process by decomposing carbon-rich organic matter to acquire carbon sources, thereby promoting polysaccharide synthesis [25]. Different cultivation formulations selectively influenced amino acid metabolism and changed the amino acid composition of Morchella fruiting bodies. Across all treatments, yield and amino acid content were not fully synchronized. Although most formulations suppressed mushroom yield, the wa-60 formula achieved a significant yield increase of 28.6% while maintaining a high total amino acid content of 19.8 g/100 g. The superior yield performance of the wa-60 formulation likely stems from the specific chemical composition of alfalfa compared to other amendments. Alfalfa has a relatively lower C/N ratio than rapeseed straw or oat straw, which may provide a more balanced carbon-to-nitrogen supply for Morchella mycelium during the critical transition from vegetative growth to primordium formation. These findings demonstrate that tailored formulation can effectively balance production and nutritional value, with wa-60 emerging as a promising cultivation strategy. The improvement in the nutritional quality of *Morchella* spp. following the addition of straw and forage further supports this conclusion.

### 4.2. Effects of ENB Formulations on Soil Fertility, Microbial Biomass, and Enzyme Activities

The application of agricultural waste can significantly alter the physicochemical properties of Morchella cultivation soil. Following tomato cultivation, increases in substrate pH and electrical conductivity (EC) promote the accumulation of nitrogen, phosphorus, potassium, and base cations in the soil [26]. Exogenous nutrient bags enhanced soil through a dual-phase pattern: initially (around 60 days), they rapidly increased key nutrients, including soil organic matter (SOM), nitrogen (N), phosphorus (P), and potassium (K), and improved soil properties; over the longer term (up to 90 days), nutrient levels remained elevated above baseline with a stable pH, confirming sustained effects. These changes directly stimulated microbial growth, reflected in rising microbial biomass carbon (MBC) and nitrogen (MBN), while microbial biomass phosphorus (MBP) exhibited a less pronounced response. Key enzymes responded with distinct timing and direction: urease (UR) activity peaked during the initial nutrient release phase, facilitating nitrogen conversion; alkaline phosphatase (ALP) activity rose alongside MBC, indicating more efficient phosphorus cycling; protease (PA) showed a delayed increase, whereas catalase (CAT) remained relatively stable across treatments. These changes result from a complex interplay of mechanisms—notably faster nitrogen transformations and stronger microbial nitrification/mineralization. However, the temporal trajectories of these variables were not entirely consistent: while SOM, N, and P generally increased over time, pH remained stable after the initial rise, and the responses of MBP and CAT diverged from other indicators, pointing to decoupled regulatory pathways. To fully understand this system, future work must reveal how specific physicochemical factors shape the activity and interactions of critical microorganisms.

### 4.3. Responses of Soil Bacterial and Fungal Communities and Their Associations with Soil Properties

The addition of agricultural waste altered the composition and diversity of soil bacterial communities, as reflected in both α- and β-diversity indices. At the phylum level, *Pseudomonadota* and *Bacteroidota* were significantly affected, while *Actinomycetota* played a positive role by driving organic matter degradation and nutrient cycling while inhibiting plant pathogen transmission [27]. *Gemmatimonadota* contributed to nitrogen/carbon cycling and fertility enhancement [28]. Different amendment formulations led to divergent bacterial community structures, with treatments such as wca-30 and wg-60 showing distinct separation from other groups. For fungi, reduced diversity may lessen competitive pressure and favor *Morchella* growth [29]. Despite treatment-induced structural differences, *Ascomycota* and *Mortierellomycota* remained the dominant phyla. *Trichoderma* was identified as a commonly dominant genus associated with fungal pathogen dynamics [30]. Correlation analyses linked specific taxa to soil properties: *Devosia* positively correlated with alkaline phosphatase, protease, MBC, nitrate-N, and SOM, while *Sphingomonas* was inversely correlated with alkaline phosphatase, protease, urease, AP, AN, and TN. *Paecilomyces* showed negative correlations with alkaline phosphatase, protease, urease, MBC, MBN, pH, and AN. Overall, the primary mechanism by which agricultural waste modifies bacterial communities is through shifts in soil physicochemical properties, with a secondary effect mediated through fungal community changes.

### 4.4. Integrated Implications, Limitations, and Practical Relevance

Overall, different formulations exhibit differentiated superiorities. The wa-60 formulation enhanced soil fertility (as indicated by higher SOM and available N), which in turn stimulated microbial biomass (increased MBC and MBN) and enzyme activities (particularly urease and alkaline phosphatase). These biochemical changes created a more favorable rhizosphere environment that selectively enriched beneficial taxa (*Bacteroidota* and *Mortierellomycota*), which are known for their roles in organic matter decomposition and nutrient mineralization. Although the wr-60 treatment (60% rapeseed straw) significantly increased soil bacterial diversity—particularly of taxa involved in lignocellulose degradation—this did not translate into higher mushroom yields. This apparent trade-off may be attributed to the relatively high C/N ratio of rapeseed straw, which likely stimulated microbial proliferation at the expense of nitrogen availability for the morel mycelium, thereby limiting fruiting body formation. Several limitations should be noted: the study was greenhouse-based, community data are compositional and correlation-based (not causal), and functional mechanisms remain unverified. Future work should validate these findings under field conditions and with functional assays. We acknowledge that our findings are limited by the controlled greenhouse conditions, single site (Jianzha County), sandy loam soil, small plot size, and one-season trial. Field-scale validation across locations, soil types, and seasons is required before recommending wa-60 to commercial growers.

## 5. Conclusions

This study investigated the effects of different organic amendment formulations on Morchella yield, amino acid nutritional quality, soil physicochemical properties, and bacterial community diversity. Based on the results of this study, several preliminary recommendations can be made for successful Morchella cultivation in the Qinghai Plateau region: (1) the wa-60 formulation (30% wheat + 60% alfalfa) is strongly recommended as a cost-effective and high-yielding ENB formula, achieving a 28.6% yield increase over the control; (2) growers are advised to monitor soil urease and alkaline phosphatase activities during the cultivation cycle, as these enzymes serve as reliable indicators of nitrogen and phosphorus availability; and (3) the enrichment of *Bacteroidota* and *Mortierellomycota* observed in the wa-60 treatment suggests that management practices favoring these microbial taxa may contribute to improved soil fertility and mushroom productivity. Notably, these physicochemical, microbial, and enzymatic indices did not exhibit fully synchronized temporal trajectories; exogenous nutrient bags induced a two-phase nutrient regulation pattern, characterized by an initial rapid increase followed by sustained stability over time. Collectively, wa-60 represents a promising organic amendment strategy for Morchella cultivation, achieving optimal yield through multifactorial coordination. Future field-scale studies incorporating functional genomic approaches are needed to verify the causal contributions of key microbial taxa to nutrient release and crop performance.

## Figures and Tables

**Table 1:** Composition of the exogenous nutrient bag formulations used in the experiment.

Treatment	Wheat (%)	Corn Cob (%)	Rapeseed Straw (%)	Organic Fertilizer (%)	Alfalfa (%)	Oat Grass (%)	Quicklime (%)
ck	30	30	30	-	-	-	10
wco-30	30	30	-	30	-	-	10
wca-30	30	30	-	-	30	-	10
wcg-30	30	30	-	-	-	30	10
wcag-15	30	30	-	-	15	15	10
wr-60	30	-	60	-	-	-	10
wo-60	30	-	-	60	-	-	10
wa-60	30	-	-	-	60	-	10
wg-60	30	-	-	-	-	60	10
wag-30	30	-	-	-	30	30	10

**Table 2:** The effect of different formulations on soil physicochemical properties.

Time	Name	SOM (g/kg)	TN (g/kg)	TP (g/kg)	TK (g/kg)	AN (mg/kg)	AP (g/kg)	AK (mg/kg)	pH
Baseline	w-0	14.92 ± 0.23 bc	0.84 ± 0.02 ab	1.25 ± 0.03 ab	20.74 ± 0.17 a	70.47 ± 4.03 a	54.39 ± 0.91 ab	392.27 ± 11.35 cd	8.23 ± 0.13 d
38 days	ck	14.59 ± 0.37 bc	0.86 ± 0.02 de	1.07 ± 0.03 abc	20.61 ± 0.29 ab	58.22 ± 5.10 a	49.87 ± 1.86 abc	536.44 ± 12.83 a	8.66 ± 0.07 abc
	wco-30	13.82 ± 0.88 c	0.82 ± 0.02 cd	1.08 ± 0.05 bc	20.52 ± 0.37 ab	65.22 ± 9.63 a	49.05 ± 4.57 abc	510.14 ± 21.06 a	8.6 ± 0.11 bc
	wca-30	13.93 ± 0.15 c	0.77 ± 0.02 bc	1 ± 0.03 de	20.73 ± 0.14 a	68.02 ± 6.03 a	42.71 ± 0.62 de	408.66 ± 19.07 bc	8.71 ± 0.06 ab
	wcg-30	14.14 ± 0.70 c	0.86 ± 0.03 de	1.05 ± 0.04 abc	20.72 ± 0.17 a	67.43 ± 2.48 a	50.32 ± 4.69 abc	426.48 ± 26.92 bc	8.76 ± 0.17 ab
	wcag-15	14.39 ± 0.62 c	0.83 ± 0.03 bc	1.09 ± 0.04 bc	20.41 ± 0.26 ab	60.78 ± 4.15 a	47 ± 3.31 bc	440.36 ± 19.56 b	8.81 ± 0.12 a
	wr-60	12.71 ± 0.79 d	0.75 ± 0.02 e	1.07 ± 0.04 d	20.64 ± 0.19 a	58.57 ± 12.5 a	38.3 ± 2.90 d	363.39 ± 18.99 d	8.82 ± 0.06 a
	wo-60	14.95 ± 0.51 bc	0.84 ± 0.04 bc	1.17 ± 0.03 bc	20.19 ± 0.25 a	65.33 ± 12.41 a	47.71 ± 2.66 bc	403.16 ± 9.61 bc	8.59 ± 0.06 bc
	wa-60	15.7 ± 0.57 ab	0.89 ± 0.04 b	1.09 ± 0.03 bc	20.19 ± 0.07 a	65.68 ± 1.93 a	45.67 ± 0.67 bc	409.89 ± 31.7 bc	8.49 ± 0.08 c
	wg-60	16.13 ± 0.50 a	1.01 ± 0.05 a	1.16 ± 0.03 ab	20.58 ± 0.22 ab	67.43 ± 4.38 a	54.73 ± 4.64 ab	507.92 ± 18.41 a	8.26 ± 0.11 d
	wag-30	14.71 ± 0.77 bc	0.83 ± 0.05 cd	1.08 ± 0.04 de	20.59 ± 0.24 ab	59.73 ± 3.74 a	42.96 ± 1.41 de	413.77 ± 36.06 bc	8.59 ± 0.10 bc
62 days	ck	14.92 ± 0.14 abc	0.85 ± 0.01 bcd	1.11 ± 0.02 a	20.66 ± 0.06 ab	69.88 ± 4.90 a	55.78 ± 3.16 a	520 ± 8.83 a	8.77 ± 0.11 bc
	wco-30	16.16 ± 0.44 a	0.88 ± 0.02 bc	1.24 ± 0.04 a	20.69 ± 0.30 ab	66.27 ± 5.30 a	57.74 ± 2.13 a	487.52 ± 10.2 ab	8.85 ± 0.10 a
	wca-30	15.8 ± 1.26 ab	0.9 ± 0.04 b	1.18 ± 0.04 bc	20.5 ± 0.30 ab	74.57 ± 4.66 a	51.06 ± 1.11 bc	472.42 ± 2.94 ab	8.83 ± 0.15 a
	wcg-30	14.21 ± 0.36 c	0.8 ± 0.01 d	1.11 ± 0.06 de	20.23 ± 0.09 ab	63.17 ± 5.15 a	46.64 ± 1.46 de	409.98 ± 7.71 de	8.92 ± 0.08 a
	wcag-15	14.56 ± 0.55 bc	0.8 ± 0.01 cd	1.18 ± 0.02 cd	20.38 ± 0.49 ab	70.07 ± 0.56 a	50.23 ± 3.2 cd	447.8 ± 6.40 c	8.93 ± 0.08 a
	wr-60	14.75 ± 0.53 bc	0.81 ± 0.03 d	1.2 ± 0.01 de	20.29 ± 0.15 ab	55.93 ± 3.68 a	46.32 ± 1.12 de	321.16 ± 5.82 e	8.65 ± 0.11 b
	wo-60	15.7 ± 0.94 ab	0.89 ± 0.05 bc	1.12 ± 0.04 e	20.18 ± 0.08 b	66.5 ± 2.52 a	44.81 ± 2.70 e	442.86 ± 6.04 bc	8.33 ± 0.11 c
	wa-60	15.39 ± 0.05 abc	0.83 ± 0.01 cd	1.14 ± 0.03 e	20.46 ± 0.27 ab	71.52 ± 3.25 a	42.73 ± 3.17 e	385.69 ± 6.62 de	8.62 ± 0.02 b
	wg-60	15.45 ± 0.82 abc	0.99 ± 0.06 a	1.11 ± 0.04 e	20.59 ± 0.42 ab	57.38 ± 3.32 a	46.06 ± 1.66 e	418.6 ± 8.90 cd	8.17 ± 0.07 c
	wag-30	15.05 ± 0.58 abc	0.96 ± 0.02 a	1.08 ± 0.04 e	20.6 ± 0.31 ab	67.15 ± 5.08 a	43.61 ± 1.92 e	408.01 ± 5.48 ab	8.19 ± 0.06 c
137 days	ck	15.34 ± 0.62 c	0.89 ± 0.09 ab	1.02 ± 0.01 b	20.61 ± 0.16 ab	63.75 ± 2.53 ab	49.05 ± 1.21 b	423.28 ± 5.77 a	8.51 ± 0.09 a
	wco-30	16.76 ± 0.87 ab	0.88 ± 0.03 ab	1.08 ± 0.01 b	20.47 ± 0.29 ab	65.45 ± 5.46 abc	48.67 ± 2.43 b	442.87 ± 10.7 a	8.52 ± 0.07 a
	wca-30	16.45 ± 0.82 ab	0.86 ± 0.03 ab	1.05 ± 0.03 d	20.39 ± 0.36 ab	64.17 ± 0.53 abc	43.38 ± 1.62 d	373.4 ± 8.54 c	8.5 ± 0.03 a
	wcg-30	15.33 ± 0.68 c	0.83 ± 0.01 b	1.1 ± 0.05 d	20.3 ± 0.13 ab	56.12 ± 3.68 de	43.56 ± 3.16 d	377.84 ± 3.35 c	8.26 ± 0.03 b
	wcag-15	16.06 ± 0.01 bc	0.88 ± 0.01 ab	1.09 ± 0.02 d	20.41 ± 0.26 ab	58.57 ± 4.18 cde	41.93 ± 1.03 d	406.52 ± 4.13 b	8.39 ± 0.04 a
	wr-60	15.69 ± 0.86 bc	0.88 ± 0.03 ab	1.15 ± 0.02 d	20.23 ± 0.15 b	61.72 ± 3.74 bcd	42.87 ± 2.80 d	437.74 ± 7.25 a	8.41 ± 0.09 a
	wo-60	15.55 ± 0.44 c	0.83 ± 0.03 bc	1.1 ± 0.03 e	20.39 ± 0.25 ab	55.88 ± 2.28 de	38.05 ± 0.91 e	429.43 ± 1.89 a	8.51 ± 0.07 a
	wa-60	17.25 ± 0.37 a	0.91 ± 0.02 a	1.15 ± 0.01 cd	20.47 ± 0.10 ab	63.47 ± 4.04 abcd	44.42 ± 1.25 cd	407.27 ± 4.97 b	8.27 ± 0.02 b
	wg-60	16.34 ± 0.25 abc	0.89 ± 0.04 ab	1.17 ± 0.04 cd	20.54 ± 0.26 ab	59.03 ± 0.81 cde	44.66 ± 0.65 cd	406.88 ± 4.67 b	8.21 ± 0.03 b
	wag-30	16.32 ± 0.64 abc	0.87 ± 0.02 ab	1.21 ± 0.04 bc	20.34 ± 0.23 ab	52.97 ± 1.41 e	46.74 ± 0.44 bc	382.43 ± 4.53 bc	8.22 ± 0.07 b

Note: Values are mean ± SD (*n* = 3). Different lowercase letters in the same column denote significant differences among treatments within the same sampling time (Tukey’s HSD test, *p* < 0.05).

**Table 3:** Effects of exogenous nutrient bag formulations on soil microbial biomass at different sampling times.

Time	Name	MBC (mg/kg)	MBN (mg/kg)	MBP (mg/kg)	UR (µg/g/h)	PA (µg/g/h)	APA (µg/g/h)	CAT (mL/g/h)
0 day	w-0	55.76 ± 4.46 bc	5.49 ± 0.29 e	5.79 ± 0.68 e	63.27 ± 3.24 cde	0.09 ± 0.01 bc	207.76 ± 3.35 ab	6.1 ± 0.21 bc
38 days	ck	96.24 ± 8.44 bc	8.66 ± 0.67 de	8 ± 1.91 de	62.98 ± 2.54 cd	0.1 ± 0.03 de	196.14 ± 3.93 ab	7.01 ± 0.20 a
	wco-30	107.73 ± 3.04 abc	10.2 ± 0.69 ade	9.03 ± 3.32 cde	68.69 ± 6.96 cd	0.08 ± 0.01 e	228.85 ± 3.08 ab	6.94 ± 0.17 a
	wca-30	88.12 ± 3.43 abc	8.92 ± 2.11 de	9.67 ± 2.63 bcde	73.22 ± 3.99 bc	0.1 ± 0.01 de	198.17 ± 1.38 ab	6.69 ± 0.23 ab
	wcg-30	105.42 ± 3.42 ab	15.11 ± 2.31 abc	13.4 ± 4.42 bc	91.21 ± 1.03 ab	0.16 ± 0.02 ab	226.74 ± 1.62 a	5.21 ± 0.36 d
	wcag-15	113.02 ± 9.54 ab	9.92 ± 2.38 de	9.83 ± 2.92 bcde	95.73 ± 2.95 a	0.14 ± 0 bc	197 ± 3.38 ab	6.15 ± 0.17 bc
	wr-60	113.72 ± 1.84 a	16.48 ± 5.69 a	10.12 ± 1.09 bcde	56.49 ± 1.77 de	0.1 ± 0.02 de	153.55 ± 6.44 c	5.99 ± 0.41 bc
	wo-60	116.15 ± 6.71 ab	15.69 ± 1.1 ab	17.69 ± 1.9 bcde	85.3 ± 0.89 ab	0.12 ± 0 cd	185.77 ± 3.82 c	5.77 ± 1.11 cd
	wa-60	87.73 ± 7.97 bc	9 ± 3.07 de	12.19 ± 0.81 bcd	93.19 ± 1.48 a	0.17 ± 0.03 a	225.16 ± 2.55 ab	3.99 ± 0.03 e
	wg-60	186.79 ± 5.96 a	18 ± 4.91 a	13.6 ± 1.02 de	88.13 ± 5.1 ab	0.12 ± 0.02 cd	228.95 ± 4.85 ab	3.89 ± 0.22 e
	wag-30	95.7 ± 0.71 bc	10.96 ± 0.87 bcd	13.17 ± 1.57 bc	51.21 ± 2.49 e	0.1 ± 0.01 de	171.9 ± 1.25 c	6.34 ± 0.33 abc
62 days	ck	59.47 ± 4.77 d	15.23 ± 4.28 ab	9.62 ± 2.02 bcd	65.03 ± 2.61 ab	0.11 ± 0.02 ab	231.02 ± 5.46 ab	5.36 ± 0.53 cd
	wco-30	140.21 ± 8.04 bc	12.91 ± 1.12 ab	11.99 ± 4.62 abc	57.1 ± 4.13 bcd	0.1 ± 0.01 b	205.81 ± 0.14 ab	6.7 ± 0.02 a
	wca-30	145.59 ± 3.61 abc	14.87 ± 2.57 ab	11.78 ± 2.56 abc	56.73 ± 7.81 bcd	0.11 ± 0.03 ab	237.63 ± 5.39 a	6.22 ± 0.31 ab
	wcg-30	135.2 ± 6.50 bc	12.01 ± 2.37 abc	9.46 ± 1.32 bcd	56.08 ± 2.09 cd	0.11 ± 0.01 bc	184.58 ± 6.02 e	5.85 ± 0.44 bc
	wcag-15	161.57 ± 7.10 ab	17.71 ± 5.53 a	15.43 ± 2.48 a	70.29 ± 7.31 a	0.09 ± 0.01 bc	192.98 ± 6.11 cd	5.52 ± 0.18 bc
	wr-60	106.03 ± 3.50 ab	10.29 ± 2.74 ab	7.5 ± 2.29 bcd	60.29 ± 2.74 bc	0.09 ± 0 be	198.95 ± 7.43 de	5.86 ± 0.39 bc
	wo-60	119.27 ± 8.60 ab	12.94 ± 4.42 ab	8.95 ± 1.89 bcd	58.47 ± 6.48 bc	0.13 ± 0.01 a	214.07 ± 4.52 c	4.71 ± 0.68 de
	wa-60	94.96 ± 9.95 cd	12.57 ± 1.75 de	13.5 ± 2.36 ab	59.43 ± 1.89 bc	0.1 ± 0.01 b	199.74 ± 1.87 cd	5.53 ± 0.79 bc
	wg-60	159.97 ± 7.87 a	15.3 ± 6.55 ab	11.6 ± 3.13 bcd	49.62 ± 2.69 de	0.06 ± 0 e	224.83 ± 4.61 ab	3.98 ± 0.34 ef
	wag-30	106.71 ± 5.97 bc	9.37 ± 2.38 bc	9.29 ± 0.66 bcd	45.27 ± 3.29 e	0.07 ± 0.02 cd	200.57 ± 2.46 cde	3.84 ± 0.34 ef
137 days	ck	201.69 ± 10.42 c	13.58 ± 0.95 ab	6.04 ± 2.0 bc	63.1 ± 0.89 c	0.11 ± 0.01 ab	294.25 ± 2.82 ab	5.66 ± 0.29 cde
	wco-30	224.37 ± 3.64 bc	15.43 ± 2.09 ab	4.15 ± 0.81 bc	48.38 ± 2.10 ef	0.11 ± 0.02 ab	238.23 ± 1.72 ab	6.67 ± 0.18 a
	wca-30	202.98 ± 5.97 c	15.91 ± 0.97 ab	10.9 ± 3.02 ab	44.32 ± 3.35 ef	0.09 ± 0.02 bc	276.25 ± 8.8 bc	6.59 ± 0.35 a
	wcg-30	235.23 ± 0.14 abc	18.29 ± 0.72 ab	8.64 ± 3.4 ab	67.09 ± 0.69 cd	0.07 ± 0.01 d	228.99 ± 6.97 de	5.82 ± 0.08 bcd
	wcag-15	381.42 ± 3.18 a	21.21 ± 3.24 a	7.71 ± 2.96 abc	85.86 ± 2.32 a	0.09 ± 0.01 bc	267.28 ± 9.04 bcd	4.68 ± 0.06 f
	wr-60	275.75 ± 7.21 ab	16.03 ± 3.81 ab	7.39 ± 0.5 abc	61.18 ± 0.43 c	0.12 ± 0.02 a	273.12 ± 5.57 ab	6.05 ± 0.15 bc
	wo-60	257.1 ± 5.16 abc	16.97 ± 1.91 ab	11.7 ± 2.42 a	62.62 ± 3.78 c	0.09 ± 0 bc	247.56 ± 5.57 bc	5.33 ± 0.31 e
	wa-60	273.94 ± 3.25 a	17.1 ± 3.10 de	9.13 ± 0.35 ab	71.15 ± 3.36 b	0.13 ± 0.01 a	322.55 ± 5.66 a	4.75 ± 0.21 f
	wg-60	216.3 ± 13.70 bc	13 ± 0.86 bc	9.13 ± 1.93 bc	54.15 ± 3.40 de	0.08 ± 0.01 cd	295.56 ± 0.86 abc	5.44 ± 0.13 de
	wag-30	190.74 ± 9.99 c	9.15 ± 2.07 cd	7.11 ± 1.02 bc	42.51 ± 1.62 ef	0.07 ± 0 d	247.82 ± 1.42 cd	5.39 ± 0.33 e

Note: Values are mean ± SD (*n* = 3). Different lowercase letters in the same column denote significant differences among treatments within the same sampling time (Tukey’s HSD test, *p* < 0.05).

**Table 4:** Groupwise comparison of alpha diversity.

Group	16S				ITS			
	Sobs	Shannon	Simpson	Chao	Sobs	Shannon	Simpson	Chao
ck	7242.67 ± 356.2 cd	11.37 ± 0.28 ef	1.00 ± 0.00	7242.67 ± 356.2 cd	472.33 ± 24.5 b	5.85 ± 0.28 a	0.96 ± 0.02 a	472.33 ± 24.5 b
wa-60	4831.67 ± 412.5 g	9.67 ± 0.35 g	0.99 ± 0.01	4831.67 ± 412.5 g	341.00 ± 28.3 d	3.97 ± 0.35 c	0.73 ± 0.04 c	341.00 ± 28.3 d
wag-30	8181.00 ± 398.7 ab	11.63 ± 0.26 ab	1.00 ± 0.00	8181.00 ± 398.7 ab	382.00 ± 22.1 c	4.85 ± 0.31 b	0.87 ± 0.03 b	382.00 ± 22.1 c
wca-30	8292.00 ± 385.3 a	11.64 ± 0.24 ab	1.00 ± 0.00	8292.00 ± 385.3 a	430.67 ± 20.8 b	5.78 ± 0.25 a	0.95 ± 0.02 a	430.67 ± 20.8 b
wcag-15	8011.00 ± 402.1 b	11.62 ± 0.27 ab	1.00 ± 0.00	8011.00 ± 402.1 b	470.00 ± 19.6 b	5.41 ± 0.29 a	0.93 ± 0.02 a	470.00 ± 19.6 b
wcg-30	7758.00 ± 391.5 bc	11.51 ± 0.29 bcd	1.00 ± 0.00	7758.00 ± 391.5 bc	423.67 ± 21.5 b	5.83 ± 0.26 a	0.95 ± 0.02 a	423.67 ± 21.5 b
wco-30	7578.33 ± 378.9 cd	11.55 ± 0.30 bcde	1.00 ± 0.00	7578.33 ± 378.9 cd	544.33 ± 26.7 a	6.18 ± 0.22 a	0.96 ± 0.02 a	544.33 ± 26.7 a
wg-60	7814.67 ± 388.2 bc	11.62 ± 0.25 ab	1.00 ± 0.00	7814.67 ± 388.2 bc	395.00 ± 23.8 c	4.86 ± 0.33 b	0.83 ± 0.04 b	395.00 ± 23.8 c
wo-60	7062.33 ± 365.8 de	11.31 ± 0.32 f	1.00 ± 0.00	7062.33 ± 365.8 de	336.67 ± 29.1 d	3.45 ± 0.38 c	0.76 ± 0.05 c	336.67 ± 29.1 d
wr-60	8518.33 ± 420.6 a	11.77 ± 0.23 a	1.00 ± 0.00	8518.33 ± 420.6 a	469.00 ± 18.4 b	5.81 ± 0.24 a	0.95 ± 0.02 a	469.00 ± 18.4 b

Note: Values are mean ± SD (*n* = 3). Different lowercase letters in the same column denote significant differences among treatments within the same sampling time (Tukey’s HSD test, *p* < 0.05).

## Data Availability

Data will be made available on request.
